# Tunable Neuromorphic Computing for Dynamic Multi-Timescale Sensing in Motion Recognition

**DOI:** 10.34133/cbsystems.0412

**Published:** 2025-09-30

**Authors:** Ruitong Bie, Xi Chen, Zhe Yang, Dong An, Yifei Yu, Qianyu Zhang, Ce Li, Zirui Zhang, Dingchen Wang, Jichang Yang, Songqi Wang, Binbin Cui, Dongliang Yang, Lin Hu, Zhongrui Wang, Linfeng Sun

**Affiliations:** ^1^ Centre for Quantum Physics, Key Laboratory of Advanced Optoelectronic Quantum Architecture and Measurement (MOE), School of Physics, Beijing Institute of Technology, Beijing 100081, People’s Republic of China.; ^2^ Beijing Key Lab of Nanophotonics & Ultrafine Optoelectronic Systems, School of Physics, Beijing Institute of Technology, Beijing 100081, China.; ^3^ Department of Electrical and Electronic Engineering, University of Hong Kong, Hong Kong, China.; ^4^ School of Microelectronics, Southern University of Science and Technology, Shenzhen, China.

## Abstract

Motion recognition, especially the distinction between high-speed and low-speed movements, is a challenging computational task that typically requires substantial resources. The extensive response range required to detect such variations in speed often exceeds the capabilities of traditional CMOS technology. This report introduces a SnS_2_-based in-sensor reservoir that offers an effective solution for detecting a variety of motion types at sensory terminals. By leveraging in-sensor reservoir computing, the device excels at classifying different motions across a wide velocity spectrum, providing a novel and promising method for motion recognition. The conductance of SnS_2_ channel under light stimulation is governed by the trapping and recombination of photogenerated carriers at the inherent defect states, which contributes to the flexible optically dynamical sensing function of the device to varying illumination times. These attributes make the device versatile for both optical sensing and synaptic emulation. The findings suggest that such a SnS_2_-based device could be instrumental in advancing motion recognition capabilities for developing next-generation artificial intelligence systems.

## Introduction

Real-time learning of motion recognition is a vital and burgeoning field within computer vision, providing the necessary intelligence for machines to decode and comprehend the intricate patterns of movement in their surroundings. This capability is not simply advantageous but rather crucial in a multitude of sectors. The technology is indispensable in applications such as autonomous vehicles, advanced robotics, and the evolving landscape of intelligent home systems [1,2].

Conventional motion recognition typically runs on digital sensors and computers using complicated machine learning models [3–6], facing challenges in both hardware and software. Hardware-wise, the performance of conventional complementary metal oxide semiconductor (CMOS) image sensors is constrained by their limited frame rate and temporal resolution, which can result in motion blur and loss of information, hindering the accurate recognition of motion dynamics. Furthermore, the requirement for a large dynamic range to discern both bright and dark details simultaneously often exceeds the capabilities of standard CMOS sensors, potentially leading to erroneous recognition outcomes. The high data volume and the necessity for greater data transmission rates and processing power for real-time motion recognition also press upon the limits of traditional CMOS sensors, necessitating supplementary strategies for data handling [7,8]. In addition, traditional edge computing systems, often based on the von Neumann architecture, are increasingly struggling with escalating energy requirements and plateauing efficiency improvements [9–11]. This struggle stems from the inherent separation of sensing, processing, and memory storage units. Software-wise, real-time training of large-scale machine learning models remains an open problem for edge devices. The conventional stochastic gradient descent involves tedious gradient computation, which is not affordable for edge devices with limited computing resources.

In-sensor reservoir computing (RC) provides a software–hardware co-design, where dynamical reservoirs exhibit states influenced by both current and recent past inputs [12,13]. Hardware-wise, in-sensor computing leverages the dynamics of sensors to process signals right where they are sensed, which collocates sensing, memory, and processing. Software-wise, RC systems offer the benefits of swift and simple training with low computational overhead. However, traditional optical in-sensor RC systems are restricted by their fixed dynamic current response to optical stimulation. As a result, the in-sensor RC systems are different to cope with motion inputs over different timescales. For example, very slow or very fast motions may not be translated to photocurrent responses that can be distinguished by the downstream processing [14,15].

To develop the in-sensor RC compatible with controllable dynamics in a wide range for motion recognition, optoelectronic memristors are urgently requested with ultra-high responsiveness, high sensitivity, and good stability [16–18]. Two-dimensional (2D) materials are considered promising candidates due to their unique structure and rich physical properties [19–30]. Recent works demonstrated retinomorphic devices based on 2D materials for motion detection [31–33], while these studies employ complex device structures and have not explored their capability to detect motion varying across different timescales. Notably, tin disulfide (SnS_2_) has a wide bandgap and high absorption coefficient, enabling superior optoelectronic capabilities. In particular, its inherent material defects or artificial defecting engineering can easily adjust its conductivity in a controllably dynamic range under electrical or optical stimulation [34], enabling the in-sensor RC with tunable dynamics in hardware.

In this study, we developed an optoelectronic in-sensor RC device based on monolayer SnS_2_ synthesized via the chemical vapor deposition (CVD) technique. This device demonstrates a notable correlation between its optic response and the duration of illumination, exhibiting excellent optical detection performance under short light illumination, such as high responsivity (R) and fast response time. Under long illumination, the sustained optic response can be used to simulate synaptic plasticity. It features tunable responsive functions, which could be leveraged for real-time edge computing like motion recognition over controllable dynamics.

## Methods

### Growth of SnS_2_ layers

The large-scale SnS_2_ nanosheets grown in this work used a dual temperature zone tube furnace (Hefei Kejing Materials Technology) under atmospheric pressure. Specifically, sulfur (S, 99.999%, Aladdin) and tin oxide (SnO_2_, 99.99%, Adamas) were precursors. Firstly, a powder mixture of 10 mg of SnO_2_ powder and 2 mg of KI (99%, Macklin) powder in a quartz boat was placed in the center of heating zone 2, and a clean Si/SiO_2_ substrate with a 285-nm SiO_2_ layer was flipped upside down on the quartz boat. Then, excess S (200 mg) was placed in another quartz boat in the center of the heating zone 1. The pure gas argon (Ar, 99.999%) was purged in the tube furnace at 500 sccm for 20 min to discharge the remaining water and air and keep an inert atmosphere inside the tube furnace before heating. Heating zone 1/2 was heated to 200 °C/700 °C within 20 min, maintained for 20 min for growth, and then slowly cooled to room temperature. The flow rate of 80 sccm of the Ar gas was used during the whole heating, holding, and cooling processes.

### Fabrication of devices

Using the wet transfer method, the SnS_2_ thin film was transferred from the original substrate onto a clean marked silicon substrate covered with 285-nm SiO_2_. Then, polymethyl methacrylate (PMMA) 950 A4 was spin-coated at the speed of 500 rpm for the first 10 s and then 4,000 rpm for the last 60 s. The sample was subsequently baked on a hot plate at 180 °C for 2 min. The sample pattern was etched by electron beam lithography (EBL) and reactive ion etching (RIE) (SHL 100μ-RIE, Beijing SHL Semi. Equipment Co. Ltd.). The patterns of source and drain electrodes were engraved using EBL with the process of development and fixing. Last, the 5/50-nm Cr/Au was deposited by an electron beam evaporator, and the unexposed PMMA and metal were peeled with acetone. Si substrate and 285-nm SiO_2_ were used as the back control electrode and dielectric, respectively.

### Electrical measurements and characterizations

The electrical and optical properties of the SnS_2_ device under various conditions were investigated using Keithley 4200A and 2636B semiconductor parameter analyzers and Lakeshore CRX-6.5K probe station. The morphology of SnS_2_ was provided by optical microscopy. The atomic structure and chemical composition of SnS_2_ were characterized using transmission electron microscopy (TEM), energy-dispersive spectrometry (EDS), and x-ray photoelectron spectroscopy (XPS). Raman spectra were recorded using a confocal Raman spectrometer with an excitation wavelength of 532 nm.

### DFT calculations

Density functional theory (DFT) calculations were performed by employing the Quantum ESPRESSO (QE) software [35,36]. The Perdew–Burke–Ernzerhof revised for solids (PBEsol) [37] was employed for computing the exchange correlation. The projected-augmented wave (PAW) method [38,39] was used, and the energy cutoff was set to 350 eV for the calculations. During atomic relaxations in crystal structure prediction, the residual forces on atoms were maintained below 0.01 eV/Å. A Monkhorst–Pack *k*-mesh of 10 × 10 × 1 was employed to discretely sample the Brillouin zone for SnS_2_ thin films. A vacuum layer of 25 Å was introduced to effectively screen the interaction between neighboring layers. Furthermore, the DFT-D3 method was incorporated to account for van der Waals corrections [40].

### Formation energies

We proceeded to examine the stability of vacancies in 2D SnS_2_. The thermodynamic stability is quantified by the formation energy (*E*_f_) and can be expressed as follows:

$$E_{f}=E_{D}-E_{P}+n\mu_{\mathrm{Sn}}+m\mu_{S}$$ (1)

Here, *E*_D_ and *E*_P_ denote the total energies of the SnS_2_ monolayer with and without vacancies, respectively, while *μ*_Sn_ and *μ*_S_ represent the chemical potentials for individual Sn and S atoms. *n* and *m* correspond to the numbers of Sn and S defect atoms relative to perfect SnS_2_. It is important to note that *μ*_Sn_ and *μ*_S_ are constrained by the relationship within the SnS_2_ monolayer [41]:

$$∆H=E\left({\mathrm{SnS}}_{2}\right)-\left[E\left(\mathrm{Sn}\right)+2E\left(S\right)\right]=\mu_{\mathrm{Sn}}+2\mu_{S}$$ (2)

Here, Δ*H* represents the formation enthalpy of SnS_2_, and *E*(SnS2), *E*(Sn), and *E*(S) denote the total energies per unit of the SnS_2_ monolayer, Sn bulk, and S molecular crystal, respectively. The energies of stable α-Sn (Fd3-m) and α-S (Fddd) at 0 K are considered.

### Conductivity calculation

The OpenMX code was employed to calculate the electrical conductivity [42], with *k*-point densities of 15 × 15 × 1 and 50 × 50 × 1 utilized for self-consistency and conductivity calculations, respectively. The code utilizes norm-conserving pseudopotential [43] and pseudo-atomic localized basis sets [44].

## Results and Discussion

### Fabrication and characterization of SnS_2_ thin films

**Fig. 1.**
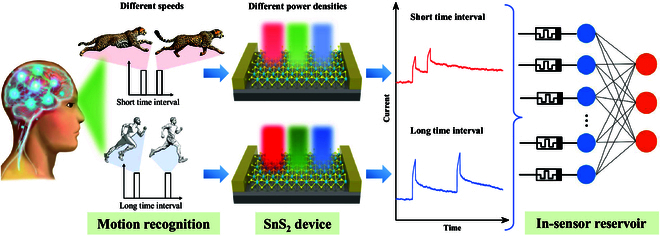
In-sensor motion recognition based on a 2D optoelectronic reservoir. Light intensity tunable dynamics of in-sensor reservoir using optoelectronic memristor. For objects moving at higher speeds, a low-intensity illumination pattern yields a fast optoelectronic response to capture fast changing spatiotemporal features. Conversely, for objects moving at a slow speed, a high-intensity light input is utilized for a slow dynamic to better adapt to the slow changing input.

**Fig. 2.**
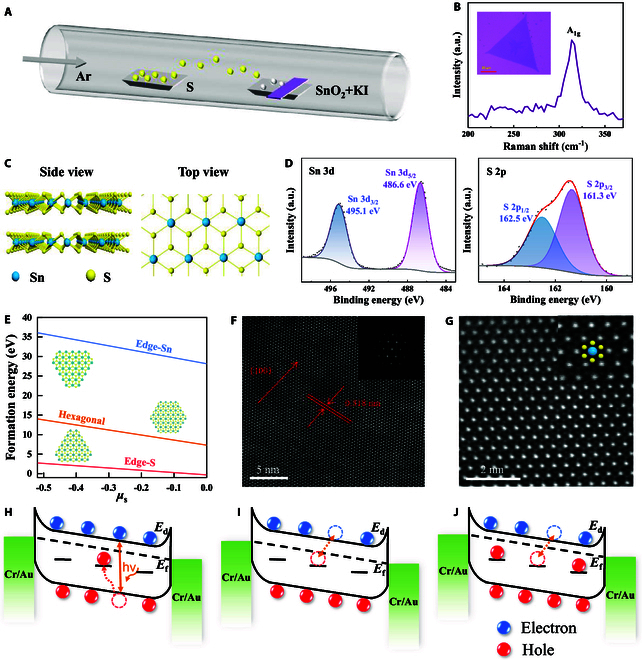
Material characterization and working mechanism of the SnS_2_ memristor. (A) Schematic diagram of the experimental setup for the CVD synthesis of SnS_2_. (B) Raman spectra of monolayer SnS_2_. The laser wavelength used is 532 nm. Inset: Optical microscope image of a typical monolayer SnS_2_ flake, whose side length of such a triangle can be around 80 μm. (C) Schematic diagram of the top and side view of the hexagonal structure of monolayer SnS_2_. (D) XPS spectra for SnS_2_: Sn 3d and S 2p. (E) The relationship diagram between formation energy and chemical potential of the grown SnS_2_ thin film indicates that a more stable structure forms a triangle shape terminated with S atoms. (F) HAADF-STEM image of SnS_2_. Inset: Corresponding fast Fourier transform pattern. (G) HAADF-STEM image of SnS_2_ shows its perfect hexagonal lattice. The inset shows the locally enlarged image of the hexagonal lattice. (H to J) Schematic of the conductance dynamics under optical stimulation with multiple timescales. (H) Electron-hole pairs are generated under light illumination, and some holes are captured by defect states induced by sulfur vacancies, leading to an upward shift of the Fermi level and an increase in channel conductance. (I) After short-duration illumination, the small number of holes occupying the defect states rapidly recombines with electrons in conduction band. (J) After long-duration illumination, a large number of holes occupy the defect states and cannot fully recombine with conduction band electrons within a short period of time.

As mentioned above, for effective motion detection with multi-timescale, it is crucial that the device should exhibit variable response characteristics to accurately detect movements at different speeds, such as high speed and low speed. If the response of the device is constant, the use of RC for classification can lead to several challenges. Specifically, objects moving at high speeds may cause the output current of the device to saturate due to the short intervals between pulses, while objects moving slowly may result in an output current that is too weak because of the longer pulse intervals. Therefore, the device must be capable of adjusting its response timescale according to the speed of the object it detects. Fig. 1 demonstrates how SnS_2_ could be employed as a motion detector, recognizing the movements of objects at varying speeds. Within this motion-detecting system, an architecture of in-sensor RC model has been utilized for classification tasks, which adopts a lower light intensity for inputs corresponding to high-speed objects, and a higher intensity for slower-moving objects. This approach enables the system to precisely discern the motion of objects across a spectrum of speeds. In this work, the 2D memristor based on our synthesized large-scale ultra-thin SnS_2_ flakes has a wide range of varying timescales for optical response, which demonstrates a hardware in-sensor RC system for such motion recognition. SnS_2_ thin film was achieved using salt-assisted CVD, as described in the methods section. The schematic diagram of the experimental setup for the CVD synthesis of SnS_2_ is shown in Fig. 2A. By introducing potassium iodide (KI) to SnO_2_, the melting point was reduced, enhancing the mass flux and reaction rate. Raman spectroscopy, performed with a 532-nm laser line as shown in Fig. 2B, reveals the characteristic peak at 313.6 cm^−1^, identified as the A_1g_ mode of SnS_2_. The inset of Fig. 2B showcases the optical microscope image of SnS_2_ flakes grown on the SiO_2_/Si substrate directly, and the side of the triangle could be around 80 μm. Moreover, the DFT calculations prove that the triangle shape of monolayer SnS_2_ terminated with S atoms is more stable due to the lower formation energy, as depicted in Fig. 2E, which is consistent with the observed statistical results on the types of shapes.

High-resolution transmission electron microscopy (HRTEM) provided further insight into the atomic structure of monolayer SnS_2_ flake. The high-angle annular dark field scanning transmission electron microscopy (HAADF-STEM) image shown in Fig. 2F displays that the lattice distance measured is 0.318 nm, assigned as the (100) plane of SnS_2_, which could match well with previous findings [45–47]. Additionally, the selected area electron diffraction (SAED) pattern inserted in Fig. 2F confirms the hexagonal structure of SnS_2_ as its monocrystalline property. Furthermore, the atomic resolution HAADF image of SnS_2_ is shown in Fig. 2G, where the intensity comparison of bright and dark atoms corresponds to Sn and S atoms, respectively. A clear enlarged image reveals the 1T phase of monolayer SnS_2_, where an Sn atom is coordinated by 6 nearest-neighbor S atoms. The top and side views of the atomic structure of 1T phase SnS_2_ are also shown in Fig. 2C. Furthermore, the chemical states of SnS_2_ are elucidated by XPS in Fig. 2D, revealing the Sn 3d and S 2p levels of SnS_2_. In particular, due to the spin-orbit coupling, the separated binding energy peak positions are at 162.5 eV for S 2p_1/2_ and 161.3 eV for S 2p_3/2_. Similarly, the binding energy peaks at 495.1 and 486.6 eV correspond to Sn 3d_3/2_ and Sn 3d_5/2_. The atomic ratio (1:1.94) of Sn to S evaluated by EDS is shown in Fig. S1, indicating the presence of S vacancies in the monolayer SnS_2_ grown by CVD. These intrinsic defects can trap and de-trap charges, modulating the conductivity of the channel in the device. Figure 2H to J depicts the internal mechanism of how the inherent defects affect the multi-timescale optoelectronic response of the device. As shown in Fig. 2H, the SnS_2_ channel generates electron-hole pairs under illumination. The photogenerated holes are subsequently captured by defect states induced by sulfur vacancies, and the trapped holes in the defect levels lead to an upward shift of the Fermi level in SnS_2_, thereby increasing the channel conductance (photogating effect). Fig. 2I illustrates the underlying mechanism of the photodetector behavior of the device under short illumination. During short light exposure, a limited number of electron-hole pairs are generated, resulting in fewer photogenerated holes being captured by defect states. Once the illumination is removed, the electrons in the conduction band quickly recombine with the small number of holes occupying the defect levels, causing a rapid decrease in the Fermi level, which manifests as a swift drop in the channel conductance. As the illumination duration increases, the recovery speed of the device conductance gradually slows down, exhibiting a photoelectric memristive behavior, as shown in Fig. 2J. Prolonged illumination generates a large number of electron-hole pairs, with a substantial portion of holes being captured by defect states and occupying deeper defect levels. This leads to a strong photogating effect, causing a substantial upward shift in the Fermi level and leading to a higher channel conductance. After the illumination is removed, the large number of holes occupying the defect states cannot quickly recombine with the channel electrons, resulting in a slow decay of the channel conductance. Consequently, the device exhibits synaptic behavior under continuous optical stimulation. The recovery time to this initial state highly depends on the duration of the illumination, which may affect the response speed of the device with a wide timescale, contributing to the accurate motion recognition of objects shown in Fig. 1.

### Optoelectronic properties of SnS_2_-based optical sensor

**Fig. 3.**
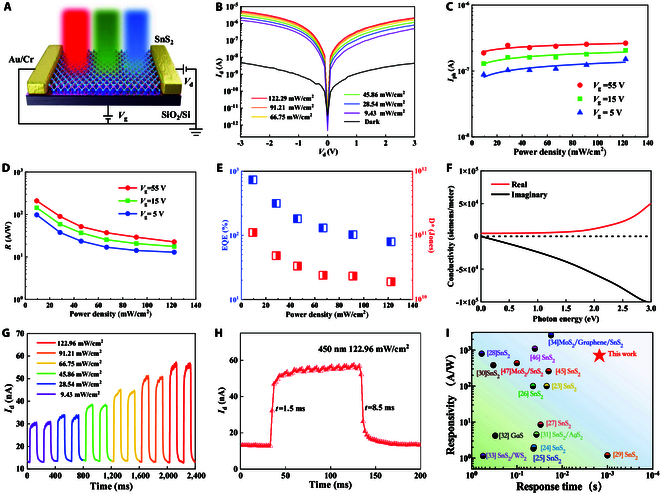
Response of optical sensor based on monolayer SnS_2_. (A) Schematic diagram of monolayer SnS_2_-based optical sensor under varied light wavelengths. (B) The output curves of the optical sensor are measured in dark and under light illumination with different power densities (λ = 450 nm). During the measurement, *V*_g_ = 0 V is applied. (C) The *I*_ph_ of the device under a read voltage of 1 V is plotted against light power densities with 3 distinct gate voltages applied. (D) Dependence of *R* on the power densities at 3 different gate voltages. (E) Power density dependent D* and *EQE* at *V*_g_ = 0 V. (F) Relationship between the real and imaginary part of conductivity and the irradiated photon energies. (G) Transient photocurrent response with the different power densities (λ = 450 nm). (H) Rise time (1.5 ms) and fall time (8.5 ms) of the light sensor at *V*_g_ = 0 V with a power density of 122.29 mW/cm^2^. (I) Comparison of responsivity and response time of this device with other previously reported results.

The optical response of the optical sensor is analyzed first. Fig. 3A shows the schematic representation of the device based on monolayer SnS_2_ exposed to different laser wavelengths. Fig. 3B presents the output curves under both dark condition and illuminated condition with varying power densities of the laser. The wavelength at 450 nm is shown here as a typical example. Fig. S2 represents its corresponding transfer curves. Notably, the photocurrent exhibits a dramatic increase as the power density is elevated. Electron-hole pairs are generated by absorbing photon energies and promptly segregated by the bias voltage. The liberated electrons and holes migrate toward the metal contacts in opposing directions, contributing an augmented light photo-response.

Additionally, the possibility of carriers captured by defect states formed from the S vacancies measured in Fig. S1 is considered. Such trapping can create an electric field effect, akin to a local gate that shifts the Fermi level, thereby mobilizing additional electrons. Consequently, carrier modulation by these trapped states may lead to a significant photoconductive gain.

Fig. 3C illustrates the correlation between photocurrent *I*_ph_ and power density. For the optical sensor based on 2D van der Waals material, the photocurrent is typically modeled by the power law relationship *I*_ph_ ∝ *P*^α^, where *P* denotes the power density and *α* is the power exponent. By analyzing the data shown in Fig. 3C, the power exponents are 0.11, 0.16, and 0.20 for different gate voltages, respectively, indicating the underlying photocurrent production mechanisms. The power exponent α close to zero suggests a photogating effect predominantly responsible for the photocurrent generation [48–50].

The key metrics of optical sensors such as responsivity (*R*), external quantum efficiency (EQE), and detectivity (D*) were then evaluated, as shown in Fig. 3D and E. The methodologies for calculating these parameters are detailed as follows:

$$I_{\mathrm{ph}}=I_{\mathrm{ill}}-I_{\text{dark}}$$ (3)

$$R=\frac{I_{\mathrm{ph}}}{\frac{P}{S_{0}}S_{1}}$$ (4)

where *I*_ill_, *I*_dark_, and *P* represent the light current, dark current, and incident laser power, respectively. *S*_0_ is the area of the laser spot, and *S*_1_ is the effective illumination area of the device.

$$\mathrm{EQE}=R\frac{\mathrm{hc}}{e\lambda }$$ (5)

$$D^{*}=R\sqrt{\frac{S_{1}}{2eI_{\text{dark}}}}$$ (6)

where *h* is Planck’s constant, *c* is the value of the light speed, *e* is the value of elementary charge, and *λ* is the wavelength of the laser. *R* decreases with the increasing power density, attributed to the fact that more photocarriers tend to recombine before being captured by the defect states under illumination with high power density. Notably, the highest *R* at *V*_g_ = 55V could reach 208.9 A/W, surpassing the previously reported SnS_2_-based photodetectors. EQE is calculated to be 733.98%, attributed to the large number of photogenerated carriers and the accumulation of charge carriers trapped by defect states. D* is impressive with values at 1.1 × 10^11^ Jones, which demonstrates its ability to detect relatively weak light signals. Figs. S3 and S4 show the same metrics for illuminating wavelengths at 520 and 650 nm. The larger *I*_ph_ observed at 450 nm indicates heightened photoconductivity at shorter wavelengths, aligning with our DFT calculations (Fig. 3F and Fig. S5).

The time-resolved optical response of the optoelectronic detection has been further investigated in Fig. 3G. Upon stimulation, the current swiftly ascends to its peak and subsequently returns to the baseline. Figure 3H reports that the rise and fall times are 1.5 ms and 8.5 ms, respectively, defined as the times for the photocurrent to rise from 10% to 90% and to fall from 90% to 10% of the peak value. In Fig. 3I, a comparative analysis of response time and responsivity is conducted, showcasing the performance of the device against counterparts utilizing different materials [34,51–64]. These findings underscore the promising potential of SnS_2_ in high responsivity for sensing applications.

### Optoelectronic properties of SnS_2_-based memristor

Besides the promising optoelectronic response of the optical sensor discussed above, one interesting phenomenon is that the same device can also show synaptic feature when the conditions of the optoelectronic pulse train vary. The device with the concurrent functions of sensing and in-memory computing enables the demonstration of in-sensor memory computing in a single device. It is well known that biological synapses facilitate the transmission of chemical signals from presynaptic to postsynaptic neurons through the release of neurotransmitters [65]. This ability to change the strength of the connections between neurons is known as synaptic plasticity, which underpins the human being’s ability to learn and remember [66–68]. In artificial synapses, the modulation of synaptic weight can be controlled by electrical and light stimuli, offering multiple degrees of freedom and paving the way for multi-perceptual systems.

**Fig. 4.**
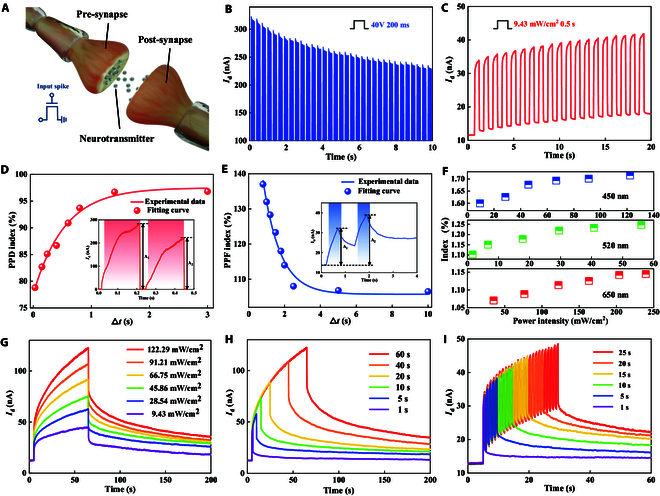
Optoelectronic response of the memristor based on monolayer SnS_2_. (A) Schematic diagram of a biological synapse with signal transmission process. (B) Current modulation by electrical pulses. A sequence of electrical pulses (the amplitude of 40 V and the width of 200 ms) with an interval of 100 ms was applied to the gate. The read voltage between the source and drain was 1 V. (C) Current modulation by optical pulses. Optical pulses were applied on the device, which was irradiated under a wavelength of 450 nm with a power intensity of 9.43 mW/cm^2^ and a pulse width of 0.5 s. The read voltage was 1 V. (D) PPD and (E) PPF indexes as a function of the time interval between 2 consecutive electrical and optical pulses, respectively. The amplitude and the width of electrical pulse trains are 40 V and 200 ms, while the power density and the width of optical pulses are 122.29 mW/cm^2^ and 0.5 s. The definition of postsynaptic current (PSC) is triggered by a pair of presynaptic spikes with a specific time interval, as shown in the inset in (D) and (E). (F) Photocurrent evolution of 2D memristor irradiated with different wavelengths at 450, 520, and 650 nm. The transitions from STP to LTP are achieved through the response of photocurrent to varied (G) power densities, (H) durations of irradiation, and (I) pulse number of the light with the wavelength of 450 nm as an example.

As shown in Fig. 4A, the synaptic plasticity could be displayed well within a 3-terminal transistor utilizing the same monolayer SnS_2_ flake as a channel material. The gate electrode and the device channel represent the presynaptic and postsynaptic membranes, respectively. Fig. 4B portrays the depression of nonlinear conductivity, modulated by electrical impulses with an amplitude of 40 V and a width of 200 ms. This is because the positive gate voltage causes the channel energy band to bend downward, making it easier for electrons in the conduction band to recombine with holes trapped in the defect states, thereby gradually weakening the photogating effect.

Furthermore, Fig. 4C illustrates conductivity facilitation achieved through successive optical pulses (9.43 mW/cm^2^ for 0.5 s). Such concurrent modulation on the conductivity of the memristor via electrical and optical pulse trains suggests that presynaptic stimulation can successively modulate synaptic weights and boost synaptic transmission efficiency in dual mode.

Paired-pulse facilitation (PPF) and paired-pulse depression (PPD), as typical characteristics of short-term plasticity (STP) for synaptic computing, represent the accumulative effect of presynaptic stimuli on postsynaptic current (PSC). The facilitation/depression effect of PSC decreases with the increasing interval time between 2 consecutive pulses. Fig. 4D shows the PPD index as a function of time intervals stimulated by 2 consecutive electrical pulses. The PPD index is defined as *A*_2_/*A*_1_, where *A*_1_ and *A*_2_ are the peak amplitudes of the PSCs generated by 2 consecutive presynaptic pulses, respectively (as shown in the inset of Fig. 4D). As shown in Fig. 4D, the PPD index increases from 78% and gradually approaches 100% with the time interval continuously increasing, indicating that the depression effect is gradually weakening. The solid red line represents the result fitted by the double-exponential decay function, which can be described as

$$\frac{A_{2}}{A_{1}}=C_{1}\cdot e^{\left(-\frac{∆t}{t_{1}}\right)}+C_{2}\cdot e^{\left(-\frac{∆t}{t_{2}}\right)}$$ (7)

where *C*_1_ and *C*_2_ are the initial amplitudes of the fast and slow phases, respectively. *t*_1_ and *t*_2_ are the corresponding characteristic relaxation times of the 2 phases. Moreover, we obtained the correlation between the PPF index and time intervals using a 450-nm laser line, as shown in Fig. 4E. The solid blue line represents the fitting results, which satisfies the double exponential decay function (as shown in the above equation). The results show that PPF index can reach its maximum value of 137% at the minimum time interval of 2 successive optical pulses.

Furthermore, the influence of the wavelength used for the generation of optical pulse trains on synaptic weights has been explored in Fig. 4F. The photocurrent evolution of the 2D memristor signifies that the ratios of the post-stimulation current to the initial current all increase under irradiation with different wavelengths of 450, 520, and 650 nm, respectively. Notably, the enhancement of photocurrent is more pronounced under the irradiation at the wavelength of 450-nm, matching well with the calculation results shown in Fig. 3F on the correlation between photoconductivity and photon energy. The original data for Fig. 4F are presented in Fig. S6 for reference. Here, the laser wavelength at 450 nm was used to effectively further evaluate the synaptic characteristics. As illustrated in Fig. 4G, with increasing light intensity, the PSC has a corresponding rise, attributed to the generation of charge carriers and the enhanced photogating effect. Higher power densities could result in extended retention times, thus effectively mimicking the transition from STP to long-term plasticity (LTP). Moreover, such a transition from STP to LTP can also be altered by varying the duration time (Fig. 4H) and the number of pulse trains (Fig. 4I), which is the result of more captured photogenerated carriers that require a longer time to recombine. Therefore, such a light-modulated memristive behavior of the device could be controlled accurately to represent varying timescales of dynamics in photo-response. Moreover, the stability of the SnS_2_ device is shown in Fig. S7. At different temperatures and humidity conditions, the device exhibits stable electric response and LTP synaptic behavior. The measurement of cycle stability in Fig. S8 shows that after 500 consecutive light pulse stimulations, the device still maintains excellent LTP synaptic performance. Fig. S9 shows the photoelectric response of 3 different batches of devices, confirming the repeatability of the SnS_2_ device.

### Dynamic real-time motion classification using in-sensor RC

**Fig. 5.**
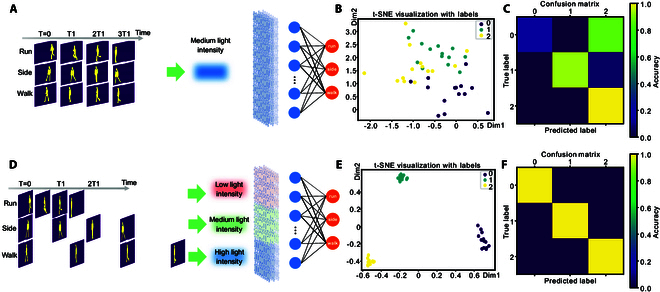
2D memristor with tunable dynamics for in-sensor multi-timescale motion recognition. (A) Video frames from the Weizmann dataset are processed into equal time segments, with a medium-intensity light source as the input for the reservoir computing layer. Post-reservoir, a trainable MLP finalizes the classification of the observed actions. (B) Visualization of the reservoir layer outputs using t-SNE for the scenario in (A), and 0, 1, and 2 represent run, side, and walk, respectively. (C) Confusion matrix depicting classification accuracy post-training for scenario (A), and 0, 1, and 2 represent run, side, and walk, respectively. (D) Video frames from the Weizmann dataset are sampled at regular intervals, with variable light intensity modulating the memristor based on the data input rate. High-frequency pulse data, such as running, employ a dimmer light to prevent saturation of the device’s output current, whereas low-frequency activities like walking use a brighter light to enhance the device output. (E) t-SNE visualization corresponding to the reservoir outputs in scenario (D), and 0, 1, and 2 represent run, side, and walk, respectively. (F) Confusion matrix showing the training results analogous to scenario (A), and 0, 1, and 2 represent run, side, and walk, respectively.

Fig. 5 showcases the real-time learning motions of different timescales using in-sensor RC, considering noise as shown in Figs. S10 and S11. Conventional in-sensor RC devices feature fixed and simple dynamic photocurrent responses. Such devices fail to recognize motion that is either too slow or too fast, as the device’s current output may become oversaturated or dissipate too rapidly, rendering the rate characteristic of the signal difficult to discern. For example, as illustrated in Fig. 5A, we have adapted 3 sample videos from the Weizmann dataset by stretching them to equal time intervals, a common practice to normalize motion videos of different timescales. These samples are then converted into medium-intensity optical pulses for input into the in-sensor RC. As the dynamics depend on the duration of motion videos, in this case, all 3 samples are subjected to the same dynamics governing photocurrent responses. The generated current from this interaction is subsequently routed to a digital circuit for further analysis. For classification purposes, the system employs a multilayer perceptron (MLP) layer.

Figure 5B displays a t-distributed stochastic neighbor embedding (t-SNE) plot that visualizes the processed data through the reservoir layer, demonstrating the ineffectiveness of this approach in distinguishing between different human motions such as running, side jumping, and walking when the timescale of all these motions is normalized. The confusion matrix presented in Fig. 5C affirms the limitations of RC in classifying when devoid of rate information.

In contrast, we leverage the tunable dynamics of the device in handling optical stimulations of different timescales. Here, we adjust the light intensity according to the speed of the object. As depicted in Fig. 5D, a weaker light is utilized as the pulse input for detecting high-speed motions, whereas a stronger light is employed for sensing low-speed motions. This adjustment ensures that the device yields optimal response outcomes for the respective speeds. The t-SNE visualization post-reservoir layer in Fig. 5E and the confusion matrix in Fig. 5F corroborate the effectiveness of this approach. By adopting this strategy, the system’s classification capabilities can be significantly improved without altering the original temporal scale of the data. The comparison of the classification accuracy of the MNIST dataset with and without considering noise is shown in Fig. S12. More details of MLP training strategy, experimental comparison of computational overhead, and noise robustness and modeling in the in-sensor RC system are discussed in Notes S1 to S3. The difference between this paper and other previous works is listed in Table S1.

From Table 1, it can be observed that the multi-timescale RC model used in this study exhibits significant advantages when applied to datasets with large temporal spans, highlighting its ability to effectively capture long-term dependencies. In comparison, other networks with a similar number of parameters, such as single-layer artificial neural network (ANN), single-layer convolutional neural network (CNN), and standard recurrent neural network (RNN) with only 4 hidden dimensions, struggle to achieve competitive performance on these types of datasets, showcasing their limitations in handling complex temporal relationships. On the other hand, networks that perform better, such as VGG11 and LSTM, require parameter counts that are more than a thousand times greater than those of the RC model. This stark contrast underscores the efficiency and scalability of the RC approach, making it a highly effective choice for tasks involving large-scale temporal data.

**Table 1.** Comparison of the network architecture proposed in this work and state-of-the-art architectures

Network structure	Accuracy	Number of parameters
Reservoir computing (this work)	100%	20,739
Single-layer ANN	36.9%	20,736
Single-layer CNN	74.3%	972,499
VGG11	98.8%	59,732,339
RNN	72%	7,979,251
LSTM	100%	31,916,531

Despite the promising results demonstrated in this work, the current system still faces several limitations that merit further investigation. Firstly, the SnS_2_-based devices exhibit excellent dynamic tunability, scaling up the in-sensor reservoir array to larger areas or high-density configurations may introduce challenges related to material uniformity and fabrication reproducibility. Potential strategies include refining the CVD growth process through real-time feedback control and adopting wafer-scale fabrication techniques to ensure device homogeneity. Secondly, while our system demonstrates effectiveness on Weizmann datasets, it is not optimized for datasets with significantly higher complexity or resolution, such as the full UCF101 dataset. To address the limitation in future work, developing preprocessing techniques for high-resolution datasets to meet system constraints while preserving motion information, and scaling reservoir layers by increasing the physical dimensions of the reservoir or implementing hierarchical reservoir architectures could be the potential strategies. These considerations highlight key areas for improving the scalability and practicality of in-sensor neuromorphic systems in future applications.

## Conclusion

In summary, this work demonstrates the multi-timescale functionality of the device based on large area monolayer SnS_2_. Significantly, the device exhibits a high *R* of 208.9 A/W, *EQE* of 733.98%, and D* of 1.1 × 10^11^ Jones. As the illumination time increases, the device exhibits the deceleration of recombination processes in monolayer SnS_2_. The synaptic behaviors are successfully simulated by a sequence of light pulses, and a transition from STP to LTP by varying the power intensities is observed. Furthermore, based on the timescales of dynamics under illumination, an in-sensor RC system in hardware based on the monolayer SnS_2_ could classify the Weizmann dataset at different speeds with an accuracy of 100%, by simulating the recognition of different motion types of run, side, and walk. This work presents an effective strategy for realizing multi-timescale motion recognition with flexible dynamics within an artificial in-sensor RC system.

## Data Availability

The data and materials used to support the findings of this study are available from the corresponding author upon reasonable request.

## Supplementary Materials

Supplementary 1 Figs. S1 to S12 Table S1 Notes S1 to S3
