# Supplementary material for: Tunable Neuromorphic Computing for Dynamic Multi-Timescale Sensing in Motion Recognition
Source: Cyborg Bionic Syst. 2025 Sep 30;6:0412. doi: 10.34133/cbsystems.0412 (PMC12604526; doi:10.34133/cbsystems.0412)
Supplement: Supplementary 1 — Figs. S1 to S12 Table S1 Notes S1 to S3 [file cbsystems.0412.f1.docx]

Supplementary Materials for

**Tunable Neuromorphic Computing for Dynamic Multi-Timescale Sensing in Motion Recognition**

Ruitong Bie^1,2#^, Xi Chen^3,4#^, Zhe Yang^1,2#^, Dong An^1,2^, Yifei Yu^3,4^, Qianyu Zhang^1,2^, Ce Li^1,2^, Zirui Zhang^1,2^, Dingchen Wang^3^, Jichang Yang^3,4^, Songqi Wang^3,4^, Binbin Cui^3^, Dongliang Yang^1,2^, Lin Hu^1,2*^, Zhongrui Wang^4*^, Linfeng Sun^1,2*^

1. Centre for Quantum Physics, Key Laboratory of Advanced Optoelectronic Quantum Architecture and Measurement (MOE), School of Physics, Beijing Institute of Technology, Beijing 100081, People’s Republic of China
2. Beijing Key Lab of Nanophotonics & Ultrafine Optoelectronic Systems, School of Physics, Beijing Institute of Technology, Beijing 100081, China
3. Department of Electrical and Electronic Engineering, University of Hong Kong, Hong Kong, China
4. School of Microelectronics, Southern University of Science and Technology, Shenzhen, China.

E-mail: [hulin@bit.edu.cn](mailto:hulin@bit.edu.cn), wangzr@sustech.edu.cn, [sunlinfeng@bit.edu.cn](mailto:sunlinfeng@bit.edu.cn)

^#^These authors contributed equally to this work.

**Keywords**

Two-dimensional materials; In-sensor reservoir computing; 2D dynamic memristor; Multi-timescale sensing

**Fig. S1.** The corresponding EDS spectrum of the atomic layer SnS_2_. Inset: the atomic ratio of Sn and S is 1:1.94.

**Fig. S2.** Transfer curves of monolayer SnS_2_-based device measured in dark and under light illumination with different power densities (λ = 450 nm).

**Fig. S3.** The optical response of monolayer SnS_2_-based optical sensor under 520nm laser illumination at three different gate voltages (V_d_ = 1V). **(a)** The I_ph_ is plotted against light power densities. **(b)** Dependence of R on the power densities. **(c)** Power density dependent D*. **(d)** EQE irradiated with the different power densities.

**Fig. S4.** The optical response of monolayer SnS_2_-based optical sensor under 650nm laser illumination with three different gate voltages applied (V_d_ = 1V). **(a)** The I_ph_ is marked as the power densities under illumination conditions. **(b)** Power-dependent R. **(c)** The trend of D* as the power density increases. **(d)** EQE as a function of light power density.

**Fig. S5.** The relationship between dielectric function and photon energy.

**Fig. S6.** Enhanced optical response with the increasing light power densities irradiated with different wavelengths of **(a)** 520 nm and **(b)** 650 nm.

**Fig. S7.** The measurements of device stability under different temperatures and humidity conditions. **(a-b)** The measurements of device stability under different temperatures of 250K, 300K, and 350K. **(a)** The comparison of output curve under dark conditions. **(b)** The comparison of optical response under 20 consecutive light pulses. At different temperatures, the SnS_2_ device exhibited stable electric response and long-term plasticity (LTP) synaptic behavior. Notably, the increasing temperature enhances the thermal excitation, resulting in the improved photoelectric response of the device with rising temperature. **(c-d)** The measurements of device stability under different humidity conditions of 10% RH and 35% RH. **(c)** The comparison of output curve under dark conditions. **(d)** The comparison of optical response under 20 consecutive light pulses. The SnS_2_ device exhibited stable electric response and LTP synaptic behavior at different humidity. It is noted that the dark current and optical response of the device slightly decreases with increasing humidity. This is attributed to the adsorption of water molecules on the surface of SnS_2_, which capture photogenerated electrons. Light pulses with a wavelength of 450 nm, a pulse width of 500 ms, and a power density of 56.31 mW/cm^2^ are used in the measurement.

**Fig. S8.** The measurements of the cycle stability after 500 times of consecutive light pulse stimulations of the SnS_2_ device. Light pulses with a wavelength of 450 nm, a pulse width of 500 ms, and a power density of 56.31 mW/cm^2^ are used in the measurement.

**Fig. S9.** The performance of other different batches of SnS_2_ devices. **(a-b)** Device #1, **(c-d)** Device #2, and **(e-f)** Device #3 exhibit repeatable output curve and optical pulse response. The measurement of output curve is conducted under dark condition. Twenty consecutive light pulses with a wavelength of 450 nm, a pulse width of 500 ms, and a power density of 56.31 mW/cm^2^ are used in the measurement of optical response.

**Fig. S10.** Mean, variance, and stability of outputs corresponding to sixteen different input states when the current device performs 4-bit optical reservoir computing (RC). **(a)** Mean and variance of the outputs for sixteen different input states when the device performs optical RC. Each state is tested 10 times. The optical intensity of the input light pulses is 9.43 mW/cm^2^, the pulse width is 1 s, and the time interval between pulses is 4 s. **(b-c)** Cycle-to-cycle stability of the device performing optical RC for each input state.

**Fig. S11.** Mean, variance, and stability of outputs corresponding to sixteen different input states when the current device performs 4-bit electric RC. **(a)** Mean and variance of the outputs for sixteen different input states when the device performs electric RC. Each state is tested 10 times. The optical intensity of the input electric pulses is 40 V, the pulse width is 0.2 s, and the time interval between pulses is 0.1 s. **(b-c)** Cycle-to-cycle stability of the device performing optical RC for each input state.

**Fig. S12.** The RC system compares the classification accuracy of the MNIST dataset with and without considering noise. The accuracy without noise is slightly higher than the accuracy when noise is considered. Notably, the classification accuracy of the Weizmann dataset using the multi-time-scale approach shows little difference between considering and not considering noise. This is mainly because the multi-time-scale method effectively separates the dataset, making it difficult for noise to have a significant impact.

|  | Network Type | Noise | Dataset |
| --- | --- | --- | --- |
| Our work | Dynamic RC | YES | Weizmann dataset |
| Ref [1] | ANN | NO | Self-made |
| Ref [2] | Dynamic RC + ANN | NO | Self-made |
| Ref [3] | Static RC | YES | Self-made |
| Ref [4] | Static RC | NO | Weizmann dataset |

**Table S1.** The difference between this paper and other previous works.

The work presented in reference [1] shares similarities with this paper to some extent. Both studies differentiate between high-speed and low-speed moving objects by modifying the network state. However, a key difference lies in their approaches: reference [1] employs a three-terminal device that adjusts response speed by varying the back gate voltage, while this paper utilizes a two-terminal device that primarily regulates response speed through light intensity. Additionally, reference [1] utilizes a multi-layer artificial neural network (ANN) for classification, with a clear separation between sensing and computing, as the sensor device does not participate in calculations. In contrast, this paper adopts a reservoir computing architecture that integrates sensing and computing. Lastly, while reference [1] illustrates concepts using example images without actual dataset classification, this paper categorizes three types of Weizmann datasets and demonstrates the feasibility of the proposed computing architecture.

Additionally, the concept in reference [2] is similar to that of this article. However, reference [2] employs reservoir computing with one thousand time steps, which approximates continuous time steps. Notably, it does not account for the inherent noise of the memristor during the simulation, which significantly impacts the calculations. Currently, the academic community predominantly uses discrete values for reservoir computing, as this approach allows for direct measurement of the output signal from the device and facilitates error analysis across different devices, making simulations more aligned with real-world conditions.

**Supplementary Note 1.** MLP training strategy.

To ensure the reproducibility and transparency of the classification results presented in this work, we provide a detailed description of the training strategy used for the multilayer perceptron (MLP) readout layer in the in-sensor RC system.

The MLP was trained using the Adam optimization algorithm, which is well-suited for training neural networks due to its combination of adaptive learning rate adjustment and momentum-based updates. This method enables efficient convergence across a variety of loss surfaces. For our multi-class classification task, we employed the categorical cross-entropy loss function to measure the difference between the predicted class probabilities and the ground truth labels.

The initial learning rate was set to 0.001, and training stability was further enhanced through the use of regularization techniques. Specifically, L2 regularization was applied to the network weights to constrain their magnitude, and dropout with a rate of 0.5 was introduced in the hidden layers to reduce the risk of overfitting. The model was trained for a maximum of 100 epochs. To avoid unnecessary computation and prevent overfitting, early stopping was employed based on the validation loss, halting training if no improvement was observed over successive epochs.

The training was conducted using a batch size of 32 samples, which offers a good trade-off between training speed and stability. The dataset was partitioned into training, validation, and test subsets following a 70-15-15 split, ensuring that the evaluation metrics reflected unbiased generalization performance. The same split was maintained throughout all training and testing procedures.

This training strategy, combining robust optimization, regularization, and early stopping, contributed to the stability and accuracy of the MLP classifier and supported the reliable interpretation of the device-level temporal encoding outputs.

**Supplementary Note 2.** Experimental comparison of computational overhead

To quantitatively support the claim of low computational overhead in our in-sensor RC system, we provide a detailed comparison with a representative conventional deep learning framework commonly used for motion recognition.

In our implementation, the input consists of four consecutive grayscale video frames of size 144×144 pixels, extracted from the Weizmann dataset. These frames are directly processed by the reservoir layer, which is implemented using two-dimensional material-based optoelectronic devices. This layer inherently performs spatiotemporal encoding by leveraging device-level physical dynamics. The output from the reservoir is then passed to a lightweight MLP classifier, which maps the dynamic responses to three motion classes. The MLP contains 62,208 learnable parameters, corresponding to the input size (144×144) multiplied by the number of output classes (3).

To provide a concrete example, we compared our approach with a traditional ResNet + LSTM architecture, a common framework for motion recognition tasks. In this model, a ResNet-18 backbone processes each input frame to extract spatial features, producing a 512-dimensional feature vector per frame. A bidirectional LSTM with a hidden size of 256 and one recurrent layer is then used to capture temporal dependencies across the four-frame sequence. Finally, a fully connected layer maps the LSTM output to three motion classes. The total parameter count for ResNet-18 is approximately 11.5 million, the LSTM contributes about 1.57 million parameters, and the classification layer adds another 1,539 parameters, bringing the total to roughly 13 million parameters.

In contrast, our system avoids explicit software-based feature extraction. The reservoir processing is performed entirely at the hardware level using physical dynamics, with only the MLP requiring training. With just 62,208 parameters, our model has over 200× fewer trainable parameters than the ResNet + LSTM baseline. This drastic reduction in model complexity translates to significant savings in computation, memory usage, and energy consumption.

Moreover, the analog nature of the reservoir dynamics enables real-time temporal processing without iterative numerical operations, making the system well-suited for edge computing scenarios where latency and power are critical. These results substantiate the computational advantages of our approach and reinforce its potential for efficient, low-power deployment in real-world motion recognition applications.

**Supplementary Note 3.** Noise robustness and modeling in the in-sensor RC system

To address potential noise in real-world edge computing scenarios, we designed and evaluated the proposed in-sensor RC system with explicit consideration of both device-level and input-level noise. The system exhibits inherent robustness due to several architectural features. First, the reservoir layer, implemented using devices based on two-dimensional materials, transforms incoming optical inputs into high-dimensional spatiotemporal feature representations. This dynamic projection enhances the separation between signal and noise by emphasizing structured temporal correlations while suppressing uncorrelated fluctuations. Additionally, the dimensionality expansion enabled by the reservoir facilitates the isolation of motion-relevant features, thereby improving robustness in the presence of noise. The final classification stage, a lightweight MLP, is trained directly on noisy data, which helps it to selectively extract discriminative information from the reservoir output and to disregard noise-sensitive artifacts.

To realistically model noise during our experiments, we introduced both device noise and dataset noise into the simulation pipeline. Device noise was modeled based on known characteristics of two-dimensional material-based devices and included both thermal noise and random fluctuations in device properties, such as channel conductance. For dataset noise, we simulated imperfections typically encountered in video acquisition by adding 5% Gaussian noise to the input frames of the Weizmann dataset during both training and testing phases. This combination of physical and signal-level noise modeling ensures that our evaluation reflects practical operating conditions. Despite these perturbations, our system consistently maintained high classification accuracy, indicating that the reservoir dynamics effectively extract relevant spatiotemporal motion features while filtering out irrelevant noise.

We also acknowledge that further testing in uncontrolled real-world environments, where more complex and dynamic noise sources may be present, is essential for future validation. To enhance robustness under such conditions, several strategies can be explored. These include optimizing reservoir device parameters such as nonlinearity and interconnectivity to maximize noise tolerance without degrading feature separability. Simple preprocessing techniques, such as spatial smoothing or temporal filtering, could be used to mitigate input noise before reaching the reservoir layer. Moreover, training data can be augmented with synthetically generated noise scenarios, including background clutter, occlusions, and lighting variation, to improve generalization. The system’s existing capability to modulate optical input intensity dynamically can also be exploited to adapt to variations in noise levels. Another promising direction is the use of ensemble reservoir arrays with slight configuration differences, whose aggregated outputs may further suppress the effect of random noise.

Overall, these results and strategies indicate that the proposed in-sensor RC system is well-suited for deployment in noisy, real-world edge computing scenarios. Its demonstrated robustness makes it a promising candidate for applications such as gesture recognition in uncontrolled environments, healthcare motion monitoring in dynamic hospital settings, and surveillance systems operating under complex background and lighting conditions. This discussion has been included in the supplementary materials to further highlight the system’s potential for reliable operation under practical, noise-afflicted conditions.

**Supplementary References**

1. Chen J, Zhou Z, Kim B J, Zhou Y, Wang Z, Wan T, Yan J, Kang J, Ahn J-H, Chai Y. Optoelectronic graded neurons for bioinspired in-sensor motion perception. *Nat. Nanotechnol.* 2023;18 (8):882-888.

2. Liu K, Zhang T, Dang B, Bao L, Xu L, Cheng C, Yang Z, Huang R, Yang Y. An optoelectronic synapse based on α-In2Se3 with controllable temporal dynamics for multimode and multiscale reservoir computing. *Nat. Electron.* 2022;5 (11):761-773.

3. Sun L, Wang Z, Jiang J, Kim Y, Joo B, Zheng S, Lee S, Yu W J, Kong B-S, Yang H. In-sensor reservoir computing for language learning via two-dimensional memristors. *Sci. Adv.* 2021;7 (20):eabg1455.

4. Sun Y, Li Q, Zhu X, Liao C, Wang Y, Li Z, Liu S, Xu H, Wang W. In‐sensor reservoir computing based on optoelectronic synapse. *Adv. Intell. Syst.* 2023;5 (1):2200196.
